# Adapt VR in dental education: boosting preclinical skill and self-confidence

**DOI:** 10.1038/s41405-025-00390-0

**Published:** 2026-01-15

**Authors:** Meriam Sherif, Nahla Barakat, Abeer Hamdy, Mohamed Fouad Haridy, Hend Sayed Ahmed, Hoda Omar Fouda, Shehabeldin Saber

**Affiliations:** 1 Department of Software Engineering, Faculty of Informatics and Computer Science, The British University in Egypt, El Sherouk City, Egypt; 2 Department of Artificial Intelligence, Faculty of Informatics and Computer Science, The British University in Egypt, El Sherouk City, Egypt; 3 Conservative Dentistry Department, Faculty of Dentistry, The British University in Egypt, El Sherouk City, Egypt; 4 Conservative Dentistry Department, Faculty of Dentistry, Cairo university, Cairo, Egypt; 5 Department of Endodontics, Faculty of Dentistry, The British University in Egypt, El Sherouk City, Egypt; 6 Dental Science Research Group, Health Research Centre of Excellence, The British University, El Sherouk City, Egypt

**Keywords:** Dental foundation training, Bonded restorations

## Abstract

**Introduction:**

Preclinical dental training requires extensive feedback and repetition, which traditional manikin exercises often lack. Adapt VR is a cost-effective, immersive system that delivers interactive, adaptive training with real-time feedback.

**Methods:**

This comparative study aimed to evaluate the effectiveness of the Adapt VR system in preclinical training. A total of 126 third-year dental students were randomly assigned to an Adapt VR Group 1 (*n* = 63), acquiring VR learning experience before practicing on laboratory simulators, or a control group (*n* = 63) who started their training on simulators directly. After practising Class I and II cavity preparations, laboratory performance was scored with a standard rubric; VR participants also completed a post-training questionnaire.

**Results:**

The VR group achieved a higher mean laboratory score (6.31) than controls (3.93; *p* < 0.001). Within the VR cohort no significant difference emerged between Class I and II scores (*p* = 0.16). Simulator averages were 81.5 for Class I and 79.4 for Class II. Most VR trainees reported increased confidence and an enhanced learning experience.

**Conclusions:**

Iintegrating the Adapt VR system into preclinical dental education significantly enhances students’ skill acquisition and self-confidence compared to conventional manikin-based training.

## Introduction

Preclinical training is a critical component of dental education, providing a structured environment for the development of manual dexterity and psychomotor skills [1]. This phase supports the integration of theoretical knowledge with practical application, allowing students to engage in simulated tasks that bridge didactic learning with clinical scenarios [2]. It also upholds patient safety by enabling students to make and learn from mistakes within a controlled, risk-free setting [3].

Traditionally, preclinical training has relied on conventional methods, such as the use of extracted or synthetic teeth, which remain commonplace in dental schools [4]. However, these approaches present several limitations, including the scarcity of suitable human teeth, inconsistencies in the material properties of artificial models, a lack of standardized feedback, and restricted opportunities for repetitive practice [5].

In response to these challenges, the integration of digital technologies into dental education has introduced an innovative instructional paradigm. These tools provide interactive platforms where students can perform irreversible procedures in a safe and repeatable manner [6]. Technologies such as virtual reality (VR), augmented reality (AR), mixed reality (MR), and haptic systems—an extension of VR—have gained increasing traction in recent years. Virtual haptic simulators replicate clinical procedures and provide immediate feedback on manual performance and procedural accuracy [7]. Furthermore, VR simulators have been shown to support the development of fine motor skills and hand–eye coordination in clinical training environments [8].

Various VR simulators have been developed to facilitate dental training, such as Simodont, PerioSim, VRDental and ImmersiveTouch, among others [6, 9–11]. These platforms offer realistic simulations of clinical procedures, structured learning experiences, real-time feedback, and performance tracking that support personalized learning pathways. Nevertheless, their adoption is constrained by factors such as high initial cost, specific infrastructure requirements, and the need for active human supervision [12].

The Adapt VR system was recently introduced as a training tool for Class I and Class II cavity preparation. It incorporates a cost-effective controller-based haptic feedback algorithm, which leverages existing VR hardware to generate tactile feedback without the need for external haptic devices. The system also includes an adaptive learning model for numerical performance assessment and an error detection algorithm that delivers prompt feedback and guidance for improvement [13].

Accordingly, the present study aims to evaluate the impact of the Adapt VR system on skill acquisition and self-confidence among dental students during their preclinical training.

## Materials and methods

Ethics approval for this study protocol was awarded by research ethics committee, Faculty of Dentistry at the British University in Egypt (IRB00012490, approval number 25-028 Date: 1/8/2025).

### Sample size calculation

The sample size per group was calculated based on Cohen’s sample size calculation formula [14]

$$n=\frac{{2({Z}_{\propto /2}+{Z}_{\beta })}^{2}{\sigma }^{2}\,}{{d}^{2}}$$

Where:

- Z_α/2_ is the Z-score corresponding to the desired confidence level.
- Z_β_ is the Z-score corresponding to the desired statistical power.
- σ is the standard deviation.
- ԁ is the medium effect size.

The total calculated sample size for this study was 126 students, evenly divided into two groups: 63 students in Group 1 (Adapt VR group) and 63 students in Group 2 (Control group). This sample size was determined to provide adequate statistical power of 0.84 (80%) to detect a medium effect size of 0.5, with a confidence level corresponding to a Z-score of 1.96 for 95%.

### Inclusion criteria

Third-year dental students from the Faculty of Dentistry were invited to participate in the study. The study’s objectives and procedures were clearly explained to all potential participants. Students who volunteered were enrolled based on the following inclusion criteria: no previous exposure to haptic simulators, no prior preclinical training, completion of an equivalent theoretical curriculum, basic computer literacy, being medically fit, and not taking any regular medications. A total of 126 students consented to participate. Demographic information, including age and gender, was collected and stored electronically. Participants were randomly assigned into two equal groups, with an equal distribution of gender across both groups: Group 1 (Adapt VR group, *n* = 63), where students received training using the VR simulation framework prior to practising on laboratory simulators; and Group 2 (Control group, *n* = 63), where students commenced training directly on laboratory simulators.

### Groups randomization

Randomization was done by the restorative department admin using generating random numbers using Random Sequence Generator, Randomness and Integrity Services Ltd. Each generated random number from 1 to 63 represents the Adapt VR group and from 64 to 126 is the Control group. Each student chose a random number placed in an opaque sealed envelope that was arranged randomly by the researcher to be assigned in one of the two groups. This process is repeated for both male and female groups.

### Adapt VR dental simulation framework

**Fig. 1:**
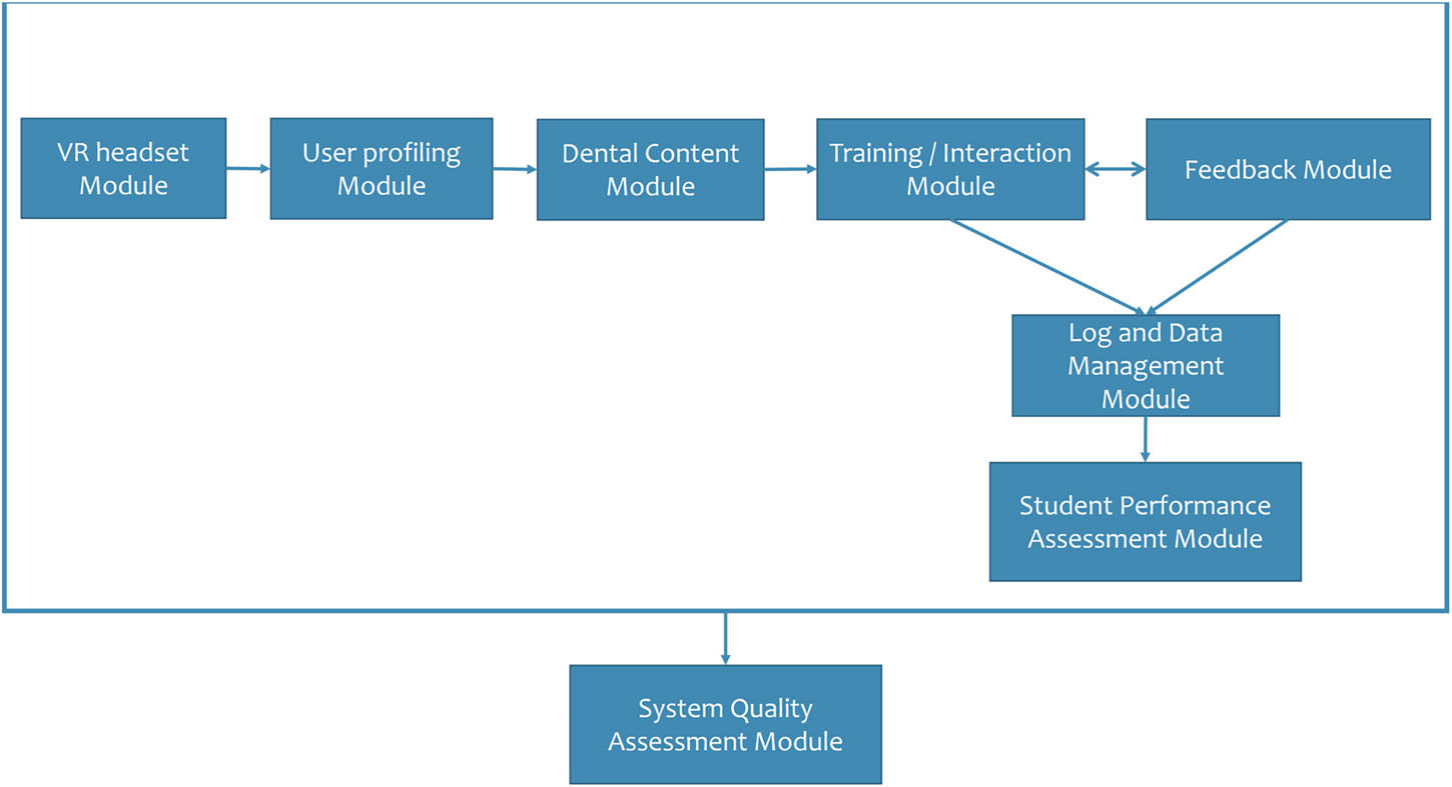
Conceptual framework for Adapt VR.

Figure 1 illustrates the Adapt VR framework, which consists of an Oculus Quest 2 VR headset (Meta Platforms Inc., Massachusetts, United States) integrated with Unity 3D IDE software (version 2021.3.10f1; Unity Technologies, Copenhagen, Denmark) [12]. The system includes a user profiling module for both students and educators, 3D models simulating a mandibular second molar, a virtual dental drilling tool, and a digital dental clinic environment. Six core algorithms were implemented to support the molar drilling simulation: collision detection, trajectory tracking, hand positioning, depth measurement, mesh deformation, and haptic feedback.

**Fig. 2:**
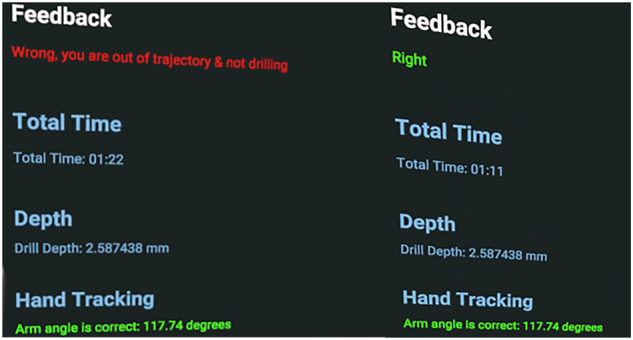
Real time feedback panel.

During training, the Oculus Quest 2 controllers served as the primary interaction tools. Each controller is equipped with an inertial measurement unit (IMU) to detect linear acceleration and rotational velocity in real time [15]. Student movements were monitored continuously via a display panel that tracked total time, drilling depth, and hand angle (Fig. 2). A green indicator signified correct alignment at a 90-degree hand angle, while red indicated deviations, improper posture, or excessive depth. The system ensured procedural accuracy by stopping the virtual drill when errors occurred and resumed operation once proper parameters were restored. The haptic feedback function was customized to deliver vibration responses relative to the force applied, enabling students to perceive tactile cues during instrumentation.

The framework also incorporates an adaptive data management module that records all student actions. If a student exits before completing a Class I procedure, their progress, log history, and profile data are stored, allowing them to resume from the point of interruption. This feature supports continuous, personalized learning. For instructors, the system generates comprehensive performance reports by aggregating student data. These reports enable educators and clinical supervisors to monitor progress, evaluate outcomes, and identify areas requiring further development.

**Fig. 3:**
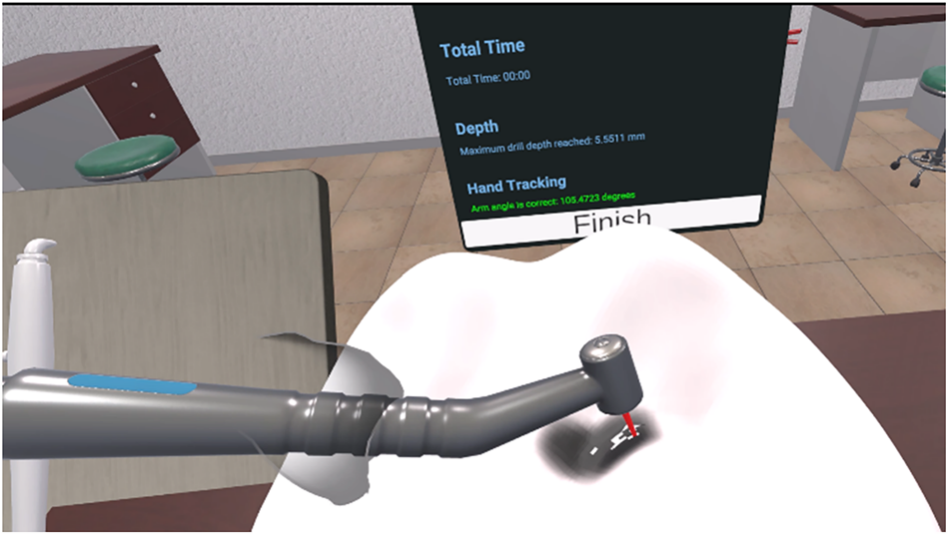
Adapt VR Simulator.

The instructions provided to all participants were as follows: Begin by practicing Class I cavity preparations on the Adapt VR dental simulator. Participants achieving a score of 65 or above proceed to Class II preparations. Those who do not reach this score must continue practicing Class I until the requirement is satisfied. Before using the Adapt VR system, the experimental group was shown a video tutorial explaining how to use the system and what they were expected to see during the simulation experience. Initially, participants receive a brief demonstration to become acquainted with the Oculus controllers and their functions. During the Adapt VR training sessions, no human supervision is provided, as the system delivers real-time feedback and guidance throughout the exercises. Figure 3 shows Adapt VR system.

### Performance metrics

The framework was specifically developed to simulate Class I and Class II cavity preparations. Students were required to begin with Class I preparation and achieve a minimum score of 65 out of 100 before progressing to Class II. If this benchmark was not attained, the student repeated the Class I procedure with a new case until the required level of competence was demonstrated. Performance was assessed using the following metrics:

- Accuracy Score (30 points): determined by dividing the number of correct actions by the total number of actions, then multiplying the result by 30.
- Efficiency Score (10 points): based on total completion time, categorised as follows: 10 points for 3–10 min (Category 1), 7 points for 1–2 min (Category 2), 5 points for 59 s (Category 3), and 0 points for durations exceeding 15 min (Category 5).
- Depth Control Score (25 points): calculated by dividing the number of over-depth errors (exceeding 2.5 mm) by the total number of actions and multiplying by 25.
- Trajectory Deviation Score (25 points): calculated by dividing the number of deviations from the planned path by the total number of actions, multiplied by 25.
- Hand Posture Score (10 points): awarded based on the system-logged hand positioning variable. A correct posture received 10 points; otherwise, a score of 0 was recorded.

**Fig. 4:**
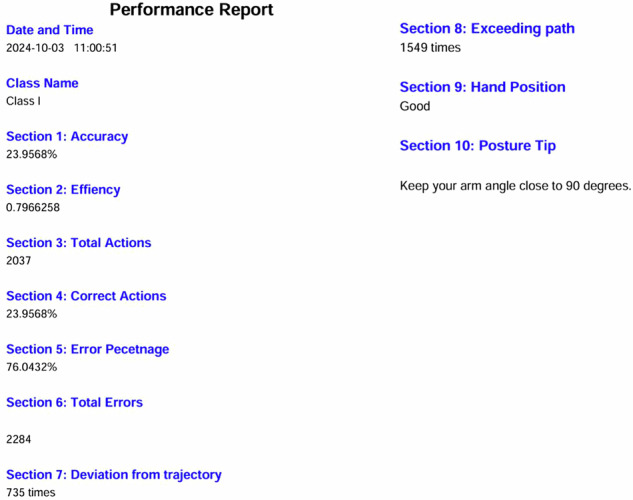
Performance Report PDF.

**Fig. 5:**
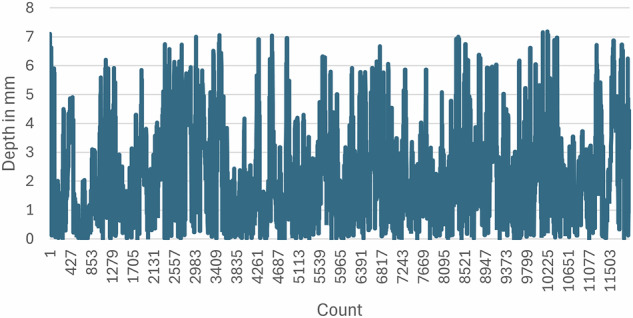
Sample Depth fluctuation in class I, for one student.

The overall score was computed from the data logged during task execution. Upon completion, a performance report was generated in PDF format and stored within each student’s profile (see Fig. 4). To further support training effectiveness, a full video recording of each session was also saved, enabling students to review their performance, track their progress, and identify areas for improvement. Figure 5 presents an example of drilling depth fluctuation (in mm) for a single student for class I, with variations between successive measurements clearly highlighted.

Following the Adapt VR simulation experience, a questionnaire employing a 5-point Likert scale was administered to students to evaluate their perceived self-confidence and learning experience (Table 1).

**Table 1:** Post VR training questionnaire assessing students’ self-confidence and learning experience.

	Strongly Agree	Agree	Neutral	Disagree	Strongly Disagree
I found the VR simulation engaging^a^					
The vibration and force (haptic feedback) give me the real feeling as if I am working by a real drilling tool^a^					
The feedback panel display provided during the simulation was helpful^a^					
The simulation helped me understand dental procedures better^b^					
I feel that my practical skills have improved after using the simulation^b^					
I am more comfortable performing dental procedures after the simulation^b^					
I am satisfied with the VR dental simulation experience^a^					
I would recommend this simulation to other students^a^					

^a^Questions assessing the learning experience.

^b^Questions assessing self-confidence.

### Laboratory simulator training

All participating students were required to perform Class I and Class II cavity preparations in the preclinical simulation laboratory using Sim Intego simulators (Dentsply Sirona, Bensheim, Germany) and artificial teeth (Nissin model; Nissin Dental Products Inc., Kyoto, Japan). The session began with a 20 min theoretical presentation introducing the learning objectives, including the outline form, preparation dimensions, and extension criteria for the cavities. This was followed by a 10 min instructional video demonstrating the complete procedure. Students then carried out the cavity preparations on Nissin teeth mounted in typodont models fixed to phantom heads to simulate clinical conditions. Practical work was conducted under direct supervision, with instructors available to provide real-time guidance when needed. The hands-on preparation lasted ~30–40 min, after which each student's work was assessed by the supervising instructor using a standardized evaluation rubric, and a score out of 10 was assigned (Table 2).

**Table 2:** Class I and Class II rubrics.

	Factors	Score out of 10
Class I	Outline	1
	Cavity Width	1
	Cavity Depth	1
	Floor	1
	Walls	1
Class II	Proximal Outline	1
	Proximal Walls	1
	Isthmus	1
	Axial wall	1
	Gingival Floor	1

## Results

The average scores achieved by students in Group 1 are summarized in Table 3. Statistical analysis using the t-test revealed no significant difference in overall scores between Class I and Class II cavity preparations within the group (*p* = 0.16209).

**Table 3:** Adapt VR dental simulation results.

Parameters	Score out of 100	Class I Results	Class II Results
Accuracy	30	13	21
Efficiency	10	5	5
Depth	25	12	22
Deviation from trajectory	25	20	21
Hand Posture	10	9.3	9.6
Total Score	100	61	79

Students’ perceptions of the Adapt VR learning experience and its influence on their self-confidence are presented in Tables 4 and 5, respectively. The majority of respondents either strongly agreed or agreed that the Adapt VR framework positively enhanced both their learning experience and self-confidence.

**Table 4:** Overall students’ perception of the Adapt VR learning experience.

	Strongly Agree	Agree	Neutral	Disagree	Strongly Disagree
I found the VR simulation engaging	18%	44.3%	24.6%	6.6%	6.6%
The vibration and force (haptic feedback) give me the real feeling as if I am working by a real drilling tool	19.7%	(54.1%)	(13.1%)	(8.2%)	(4.9%)
The feedback panel display provided during the simulation was helpful	29.5%	41%	(23%)	(4.9%)	(1.6%)
I am satisfied with the VR dental simulation experience	39.3%	41%	16.4%	3.3%	\
I would recommend this simulation to other students	29.5%	44.3%	21.3%	3.3%	1.6%

**Table 5:** Overall students’ perception on boosting self-confidence.

	Strongly Agree	Agree	Neutral	Disagree	Strongly Disagree
The simulation helped me understand dental procedures better	13.1%	36.1%	36.1%	9.8%	4.9%
I feel that my practical skills have improved after using the simulation	9.8%	23%	39.3%	16.4%	11.5%
I am more comfortable performing dental procedures after the simulation	9.8%	31.1%	41%	14.8%	3.3%

Performance outcomes on the laboratory simulator for both groups are presented in Table 6. The mean score for Group 1 (Adapt VR) across Class I and Class II cavity preparations was 6.31, whereas Group 2 (Control) achieved a mean score of 3.93 for Class I and Class II cavity preparations. Inter-group comparison revealed a statistically significant difference favouring the students who underwent prior training with the Adapt VR system (*p* < 0.001).

**Table 6:** Laboratory simulator results.

	Factors	Group 1 (AdaptVR mean)	Group 2 (Control mean)
Class I	Outline	0.67	0.39
	Cavity Width	0.57	0.54
	Cavity Depth	0.54	0.39
	Floor	0.55	0.22
	Walls	0.70	0.32
Class II	Proximal Outline	0.72	0.47
	Proximal Walls	0.70	0.60
	Isthmus	0.63	0.19
	Axial wall	0.57	0.31
	Gingival Floor	0.62	0.45

The t-test outcome revealed statistical significance between the averages of the two groups (*p* = 0.00011).

## Discussion

Contemporary dental education requires the integration of affective, cognitive, and psychomotor skills to bridge theoretical knowledge with clinical practice [16]. Digital technologies used for this purpose range from basic VR goggles and desktop haptics to fully immersive VR simulators with motion tracking and haptic feedback [17]. VR platforms offer key advantages, including enhanced spatial awareness, repetitive hands-on practice with immediate feedback, and personalized learning all without requiring continuous instructor supervision [18]. However, high initial costs limit widespread adoption in dental schools.

Third year students were selected for this study as they are in the preclinical stage of learning as they work on models that are mounted in dummy heads of simulators. They have basic knowledge about anatomy, dental instruments and dental materials. They are trained on laboratory simulators to develop a foundational set of skills and competencies that prepare them for clinical practice. Therefore, the impact of VR simulation on skill acquisition and self-confidence was assessed at this phase of curriculum implementation.

To address this, the present study introduced the Adapt VR framework, a cost-effective solution utilizing built-in VR controller capabilities for haptic feedback. The system was evaluated for its impact on students' learning experience, skill acquisition, and self-confidence during preclinical training. The study followed a VR-first approach, which aligns with Felszeghy et al. [19], who found improved manual dexterity and self-efficacy through early VR exposure. The interactive, low-stress environment enabled students to visualize procedural errors in 3D, enhancing their procedural understanding [20].

Most participants reported that the Adapt VR simulator improved both their perceived self-confidence and learning experience, consistent with findings by Gottlieb et al [21]. and Alvitez-Temoche et al. [22], who highlighted VR’s role in enhancing clinical readiness. These results partially align with Quinn et al. [23], who recognized the benefits of VR for feedback and independent learning but found conventional training more effective in boosting confidence.

In terms of skill acquisition, students who underwent VR pre-training scored significantly higher on cavity preparation assessments than those without. This supports findings from Algarni et al., [24] Lee et al. [25], and Patel et al. [26], who all reported faster and more accurate performance following VR-based training. Similarly, LeBlanc et al. [27] noted improved outcomes when VR supplemented traditional teaching. However, our results contrast with Vincent et al. [28] and Dwisaptarini et al. [29], who observed no significant differences between VR and conventional methods. Such discrepancies may be attributed to variations in simulation algorithms, training duration, or assessment criteria.

One of Adapt VR’s most impactful advantages is its ability to provide immediate, data-driven feedback on parameters such as accuracy, force application, tool trajectory, and completion time. This rapid feedback loop is crucial for skill development, as supported by Nguyen et al [30]. and Tusher et al. [31], who found that real-time feedback accelerated learning and improved performance retention.

Additionally, Adapt VR allows unlimited practice opportunities, often restricted in traditional models due to resource constraints. Nevertheless, this study faced limitations, including the absence of skill retention assessment and logistical challenges such as limited VR equipment and student scheduling conflicts, which required the study to be conducted over multiple sessions.

Despite these constraints, the findings underscore the value of Adapt VR as a supplementary educational tool in dental training. Future research should focus on standardizing VR-based protocols, refining hybrid learning models, and assessing long-term clinical outcomes and patient care competencies.

## Conclusions

This study demonstrated that integrating the Adapt VR system into preclinical dental education significantly enhances students’ skill acquisition and self-confidence compared to conventional manikin-based training. Third-year dental students who engaged with Adapt VR exhibited higher laboratory performance scores, with improvements in both procedural accuracy and consistency. These results highlight the value of real-time feedback, adaptive learning, and repeated practice offered by VR technology in developing psychomotor skills critical for dental competency. Satisfaction among users was high, and the system proved robust and user-friendly. Although the study revealed strong outcomes, it did not assess long-term retention of skills, indicating a need for future research in this area. In summary, the findings support incorporating cost-effective VR solutions like Adapt VR alongside traditional training in dental curricula, with future studies recommended to establish standardized VR protocols and evaluate their sustained benefits in clinical practice.

## Data Availability

All data available upon request from the corresponding author.
